# Characterization of the IDENTIFY^TM^ surface scanning system for radiation therapy setup on a closed‐bore linac

**DOI:** 10.1002/acm2.14326

**Published:** 2024-03-18

**Authors:** Janita Dekker, Sander van het Schip, Marion Essers, Mariska de Smet, Martijn Kusters, Willy de Kruijf

**Affiliations:** ^1^ Institute Verbeeten Tilburg Netherlands; ^2^ Radboud University Medical Center Nijmegen Netherlands

**Keywords:** accuracy, phantom measurements, surface guided radiation therapy, technical performance

## Abstract

**Purpose:**

In radiation therapy, surface guidance can be used for patient setup and intra‐fraction motion monitoring. The surface guided radiation therapy (SGRT) system from Varian Medical systems, IDENTIFY^TM^, consists of three pods, including cameras and a random pattern projector, mounted on the ceiling. The information captured by the cameras is used to make a reconstruction of the surface. The aim of the study was to assess the technical performance of this SGRT system on a closed‐bore linac.

**Methods:**

Phantom measurements were performed to assess the accuracy, precision, reproducibility and temporal stability of the system, both in align and in load position. Translations of the phantoms in lateral, longitudinal, and vertical direction, and rotations around three axes (pitch, roll and yaw) were performed with an accurate, in‐house built, positioning stage. Different phantom geometries and different surface colors were used, and various ambient light intensities were tested.

**Results:**

The accuracy of the IDENTIFY^TM^ system at the closed‐bore linac was 0.07 mm and 0.07 degrees at load position, and 0.06 mm and 0.01 degrees at align position for the white head phantom. The precision was 0.02 mm and 0.02 degrees in load position, and 0.01 mm and 0.02 degrees in align position. The accuracy for the Penta‐Guide phantom was comparable to the white head phantom, with 0.06 mm and 0.01 degrees in align position. The system was slightly less accurate for translations of the CIRS lung phantom in align position (0.20 mm, 0.05 degrees). Reproducibility measurements showed a variation of 0.02 mm in load position. Regarding the temporal stability, the maximum drift over 30 min was 0.33 mm and 0.02 degrees in load position. No effect of ambient light level on the accuracy of the IDENTIFY^TM^ system was observed. Regarding different surface colors, the accuracy of the system for a black phantom was slightly worse compared to a white surface, but not clinical relevant.

**Conclusion:**

The IDENTIFY^TM^ system can adequately be used for motion monitoring on a closed‐bore linac with submillimeter accuracy. The results of the performed measurements meet the clinical requirements described in the guidelines of the AAPM and the ESTRO.

## 1 INTRODUCTION

Optical surface scanning, or surface guided radiation therapy (SGRT), is an upcoming technique in the field of radiation therapy and is used for positioning of patients and for intra‐fractional motion management during radiation treatment. Surface scanning is a fast, non‐invasive imaging technique with a large field of view (FOV), without using ionizing radiation, making it suitable for daily setup and motion monitoring.^1^, ^2^ SGRT helps to improve the exact posture of the patient, prior to the online imaging and has already shown its benefit for various treatment sites, such as head and neck, breast, lung, and prostate.^2^, ^3^, ^4^, ^5^, ^6^ Since the external surface of the patient is visualized, online imaging still needs to be performed for many target volumes to verify the internal anatomy.^7^, ^8^

Despite a widespread use of surface scanners in recent years, clear guidelines on the use of SGRT were lacking until recently. This was the motivation for the SGRT working group of the ESTRO to give recommendations on SGRT roles and responsibilities and to specify important aspects of commissioning and quality assurance (QA) methods.^1^ The American Association of Physicists in Medicine (AAPM) also formulated recommendations on QA methods of SGRT and described the different parts of commissioning an SGRT system.^9^, ^10^

There are multiple vendors who developed commercially available optical surface scanning systems. This paper focusses on the IDENTIFY^TM^ system (Varian Medical Systems, Palo Alto, CA). The IDENTIFY^TM^ system is developed for use in radiation therapy, to position a patient, and to perform intra‐fractional motion measurements, and breath‐hold monitoring.^6^ The system comprises of three pods, each consisting of a random pattern projector using blue LED light and two stereoscopic cameras, which are mounted to the ceiling. A unique pattern of dots creates a textured projection on the surface of an object. The camera pairs capture the reflected pattern to calculate the location of each point of the pattern. The frame rate ranges between 5 and 10 frames per second, for an ROI of size 10 × 10 cm. The FOV is 40 × 100 × 40 cm (lateral, longitudinal, vertical) in diameter around the isocenter. The information of the three pods is combined to make a reconstruction of the surface. The reconstructed surface is rigidly registered to the reference surface and a 3D transformation with respect to the isocenter is calculated.^11^ Depending on the treatment volume, the registration area used to calculate the translations and rotations around the isocenter can be reduced by defining a region of interest (ROI).

When the IDENTIFY^TM^ system is applied in combination with a C‐arm linear accelerator (linac), such as a TrueBeam^TM^ system (Varian Medical Systems), one pod is mounted on the ceiling centrally located above the foot‐end of the couch, while the other two pods are mounted on the ceiling on the left and right side of the couch. A more recent application of the IDENTIFY^TM^ system is to apply it in combination with a closed‐bore linac, such as an Ethos^TM^ or Halcyon^TM^ system (Varian Medical Systems). For this application, the position of the pods is adjusted in order to prevent camera blockage by the closed‐bore linac. Hence, two pods are mounted in front at the sides of the patient on the ceiling, and the third is mounted above the head‐end of the couch on the ceiling. For Ethos and Halcyon systems, the couch positions for set‐up and for treatment differ about 58 cm in length and are called “align” and “load”, respectively. The couch is located outside the bore in align position, enabling patient positioning at “simulation isocenter” of the patient. During patient treatment, the couch is located inside the bore with the treatment isocenter at load position. So, the difference in couch position between align and load is approximately 58 cm in length plus the difference between simulation isocenter and treatment isocenter, also known as the “delta couch shift”. Hence, possible differences in surface reconstruction between both positions must be investigated.

The aim of the study was to characterize the technical performance of the SGRT system IDENTIFY^TM^ on a closed‐bore linac. A thorough analysis was performed with different phantom geometries, different skin colors, and under various ambient light conditions to establish the accuracy, precision, reproducibility, temporal stability, and localization accuracy of the system.

## 2 METHODS

### 2.1 Experimental setup

Translations of the phantoms in lateral and longitudinal direction, and rotations around three axes (pitch, roll, and yaw) were performed with an in‐house built positioning stage. The resolution of the translations of the positioning stage was 0.05 mm, as determined using a dial gauge (Mitutoyo, accuracy 0.01 mm). The angle of the positioning stage was measured with an inclinometer, having an accuracy of 0.05° (LD‐2 M Dual axis inclinometer). The translations in vertical direction were performed by the couch of the linac, since the positioning stage was not able to move vertically. The resolution of the vertical translation of the couch was 0.01 mm, as determined using a dial gauge.

Before all measurements, we followed the calibration procedure as recommended by the vendor, including a daily QA. To be able to start with the daily QA the cameras have to be thermally stable. An isocenter check of the cameras is performed by using a calibration board. The board has to be aligned with the treatment machine isocenter and the offset of the camera isocenter is calculated.

The IDENTIFY^TM^ system (version 2.3) installed at an Ethos system and a Halcyon system was used to perform the measurements. The measurements were performed in align as well as in load position. Except for the measurements investigating the effect of the ambient light level on the system accuracy, the standard ambient light level of 90 lux was applied

### 2.2 Phantoms and ROI definition

**FIGURE 1:**
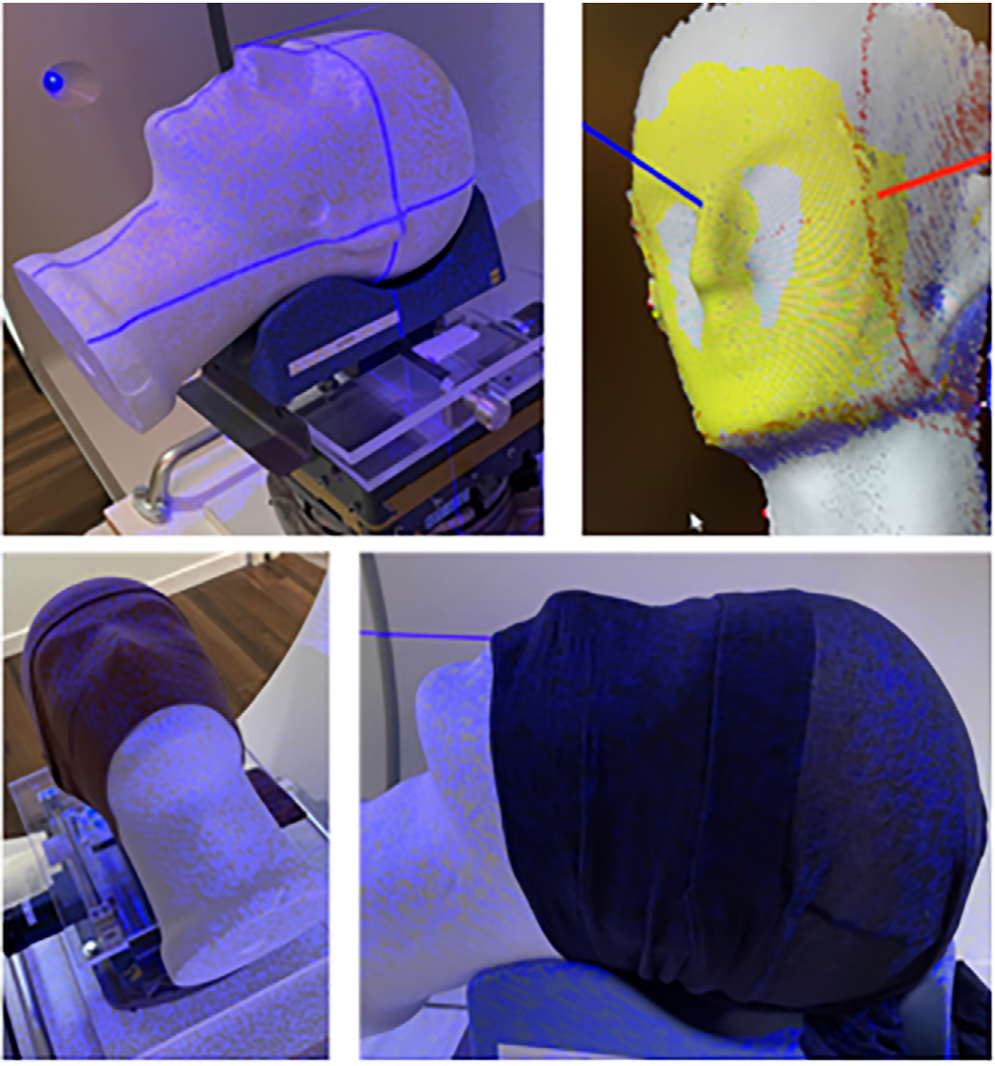
The head phantom was placed on a positioning stage. The ROI is shown in yellow. To mimic different skin colors, brown and black tights were pulled over the head phantom.

**FIGURE 2:**
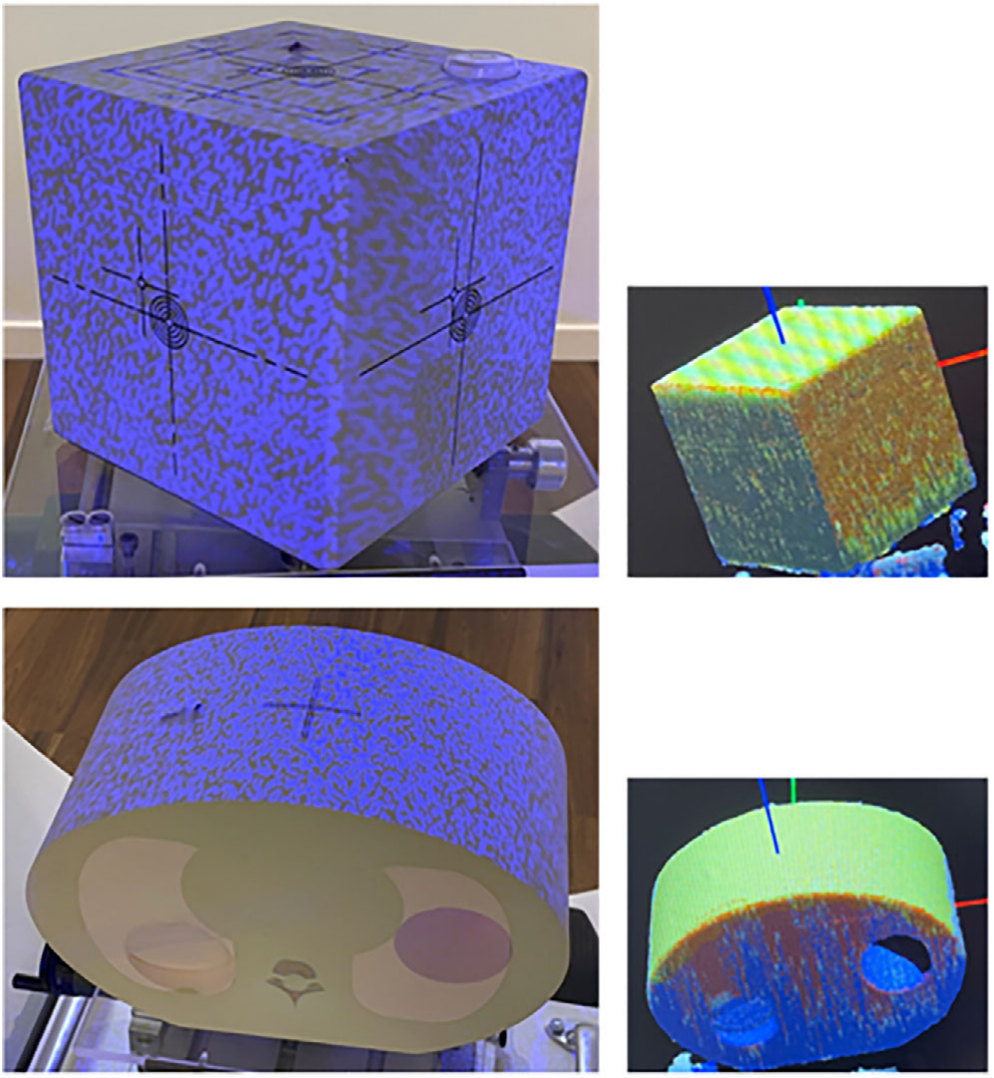
Besides the head phantom, the Penta‐Guide phantom (upper pictures), and the CIRS lung phantom (lower pictures) were used to assess the accuracy and precision of the IDENTIFY^TM^ system. The ROI is shown in yellow.

To investigate the performance of the IDENTIFY^TM^ system for different surfaces, the measurements were performed using various phantoms: a Styrofoam head phantom, the Penta‐Guide phantom (QUASAR), and the CIRS lung phantom (Figures 1 and 2). These phantoms were selected based on the difference in 6D features: the head phantom has more 3D features compared to the cubical Penta‐Guide phantom and compared to the oval shaped CIRS lung phantom.

The head phantom was placed on a standard head support (Orfit Industries, Wijnegem, Belgium). A ROI was drawn including the frontal bone, nose, maxilla, and mandible (Figure 1). The ROI of the Penta‐Guide phantom included the top side, and two front sides of the phantom (Figure 2). The ROI of the CIRS lung phantom included the top side (Figure 2).

### 2.3 Measurements

Several measurements were performed to determine the accuracy and precision of the system. The reported accuracy was relative to the starting position of the measurement. At the start of each measurement, the motion monitoring was reset, meaning that all 6 translation and rotation values were set to zero and a new reference was defined as the detected surface at time zero. The displacement of the phantom was incrementally increased in a specific direction (either translation or rotation). The translations and rotations measured by the IDENTIFY^TM^ system were recorded for 20 s at each position. Although in a clinical setting the deviations measured by the IDENTIFY^TM^ system are displayed on a screen in centimeters with two decimal places, the results in this study were based on the analysis of stored raw data in millimeters with six decimal places. The raw data was analyzed using Matlab (version 2015b, MathWorks).

The platform was moved 15 mm or 3 degrees, in steps of 0.5 mm or 0.5 degrees, respectively. This measurement was performed for translations (left, right, cranial, caudal, anterior, posterior) and rotations (pitch, roll, and yaw). To determine the accuracy and precision for very small movements additional measurements were performed for which the phantom was moved in the smallest steps that was possible with the positioning stage. The phantom was moved in steps of 0.05 mm to a translation of 0.5 mm in total. This was performed for the three translational directions. Moreover, the phantom was moved in steps of 0.05 degrees for pitch and roll, and in steps of 0.033 degrees for yaw, to 0.5 degrees in total.

Measurements were performed in load as well as in align position, for the three phantoms. Displacements in lateral, longitudinal, vertical, pitch, roll, and yaw direction were performed with the head phantom. Displacements in lateral, longitudinal, vertical, and roll direction were performed with the Penta‐Guide phantom and the CIRS lung phantom.

### 2.4 Accuracy and precision

Accuracy is the systematic offset between the average of the measurements and the true value.^12^ The accuracy at position p for a specific direction was defined as:

$$acc_{p}=\frac{1}{N}\;\sum_{i=1}^{N}\left(\left|x_{{\text{IDENTIFY}}^{\text{TM}}}-x_{true}\right|\right)$$

with N is the number of datapoints, $x_{{\text{IDENTIFY}}^{\text{TM}}}$ is the value measured by IDENTIFY^TM^, and $x_{true}$ is the applied translation or rotation. By averaging over all positions the accuracy for each direction was defined as:

$$acc=\frac{1}{P}\;\sum_{j=1}^{P}acc_{p}$$

with *P* is the number of positions.

Precision was defined as the variability of a measurement around the average value and was represented here by the standard deviation. The precision at position p was given by:

$$prec_{p}=\sqrt{\frac{\sum {\left(x_{{\text{IDENTIFY}}^{\text{TM}}}-\mu_{p}\right)}^{2}}{N}}$$

with $\mu_{p}$ is the mean value measured at position p, and N is the number of data points. By averaging over all positions the total precision was defined as:

$$prec=\frac{1}{P}\;\sum_{j=1}^{P}prec_{p}$$

### 2.5 Reproducibility

To assess the reproducibility a repeated measurement was performed. The white head phantom was moved ten times to the same position 10 mm lateral from the isocenter.

Reproducibility was defined as the standard deviation of repeated measurements:

$$repr=\sqrt{\frac{\sum {\left(\mu_{i}-\mu \right)}^{2}}{M}}$$

with M the number of repeated measurements, $\mu_{i}$ the mean value of measurement i, and *µ* the average over all measurements.

### 2.6 Temporal stability

Temporal stability is a measure for the constancy of the measurements over time. It is the ability to maintain a consistent output, when the object that is measured is not moving. A static measurement of 30 min was performed with the white head phantom placed in the isocenter to determine the temporal stability of the system.

### 2.7 Effect of surface colors on localization accuracy

The performance of the SGRT system can be influenced by the color of the surface or the skin of a patient. This was tested by using head phantoms in black (RAL color 9011), brown (RAL color 8003), and white (RAL color 9002) that mimics different skin colors (Figure 1). To realize this, black and brown tights were doubled and pulled over the white head phantom. Displacements in lateral direction were performed in load position to determine the accuracy and precision. No differences in performance are expected between align and load position, hence measurements in align position were not performed.

### 2.8 Effect of ambient light on the system accuracy

The effect of the ambient light on the performance of the IDENTIFY^TM^ system was tested using the white head phantom. Three ambient light intensities, 25 lux, 70 lux, and 90 lux, were applied and the phantom was displaced in lateral direction. To assess possible performance differences between align and load position, measurements were performed in both positions to determine the accuracy and precision.

### 2.9 End‐to‐end localization assessment

The end‐to‐end localization error was assessed using the Penta‐Guide phantom. First, a CT‐scan with slice thickness of 1.25 mm was acquired. The external body was contoured in Eclipse using the body contour tool with a threshold value of −350 Hounsfield units (HU). Next, the phantom was aligned to the laser lines in align position. The measurements were performed on two Halcyon systems. Then, the couch was moved from align to load position and a CBCT‐scan was acquired. The CBCT‐scan was matched to the CT‐scan and the couch shift was applied. For a closed‐bore linac two positions are important: align and load position. The localization error in align position equals the values measured by the IDENTIFY^TM^ system when the phantom was positioned according to the lasers.  The localization error in load position equals the values measured by the IDENTIFY^TM^ system after the couch shift was performed according to an online matched CBCT‐scan.

### 2.10 Investigation of optimal HU threshold for skin contouring

The external body contour of a patient derived from the CT‐scan is used to reconstruct the surface and serves as reference. Usually, a threshold value of −350 HU is used to align the external body contour. Errors in the alignment will result in deviations in the reference scan, causing set‐up errors. Therefore, the effect of the use of different thresholds values for the correct value of the HU used for the body contour was investigated. The distance between the different contours was measured.

## 3 RESULTS

### 3.1 Accuracy and precision

The accuracy and precision of the IDENTIFY^TM^ system for the white head phantom, the Penta‐Guide phantom, and the CIRS lung phantom is given in Table 1. The accuracy and precision were determined for the directions in which the phantom was moved. The resolution of the positioning stage is 0.05 mm and 0.05 degrees.

**TABLE 1:** Accuracy and precision of IDENTIFY^TM^, measured at load as well as align position, for displacements of the white head phantom, the Penta‐Guide phantom, and the CIRS lung phantom in lateral (lat), longitudinal (lng), vertical (vrt), pitch, roll, and yaw direction.

		Applied displacement					
Accuracy (precision)		Lat (mm)	Lng (mm)	Vrt (mm)	Pitch (°)	Roll (°)	Yaw (°)
White head phantom	Load	0.02 (0.01)	0.03 (0.01)	0.07 (0.01)	0.07 (0.01)	0.03 (0.02)	0.02 (0.01)
	Align	0.03 (0.01)	0.06 (0.01)	0.03 (0.01)	0.01 (0.01)	0.10 (0.01)	0.01 (0.01)
Penta‐Guide phantom	Load	0.02 (0.01)	0.04 (0.01)	0.06 (0.01)		0.02 (0.01)	
	Align	0.06 (0.01)	0.06 (0.01)	0.03 (0.01)		0.01 (0.01)	
CIRS lung phantom	Load	0.01 (0.04)	0.01 (0.01)	0.06 (0.01)		0.01 (0.02)	
	Align	0.20 (0.01)	0.01 (0.01)	0.06 (0.01)		0.05 (0.01)	

**FIGURE 3:**
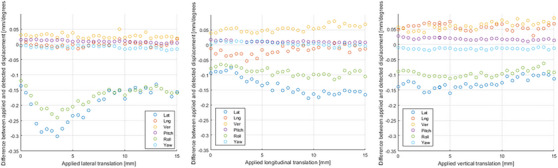
Difference between the applied and the detected translation of the CIRS lung phantom by IDENTIFY^TM^.

Figure 3 shows the difference between the applied translations and the detected movement by the IDENTIFY^TM^ system, for the CIRS lung phantom in align position. Although the system resets the reference image, a small lateral and roll displacement are present at the start of the measurements for this specific phantom. When the phantom was translated, these errors remained present or even increased. Also in load position, a lateral displacement of the phantom also resulted in a small roll rotation.

We determined the maximum value of the accuracy in a specific point for a specific measured displacement (either longitudinal, lateral, vertical, pitch, roll, or yaw) when performing any of the displacements. Table 2 gives the values of this maximum value of the point accuracy in load position as a 6 × 6 matrix. To give an example on how to interpret this table: the maximum value of the accuracy for the measured roll when a lateral displacement was performed, was 0.17 degrees.

**TABLE 2:** Maximum value of the accuracy IDENTIFY^TM^, for different phantom displacements in a specific direction, measured at load position.

		Measured deviation					
		Lat (mm)	Lng (mm)	Vrt (mm)	Pitch (°)	Roll (°)	Yaw (°)
White head phantom displacement	Lat	0.09	0.09	0.10	0.02	0.05	0.03
	Lng	0.05	0.06	0.32	0.39	0.09	0.02
	Vrt	0.09	0.22	0.12	0.03	0.05	0.06
	Pitch	0.06	0.07	0.10	0.09	0.07	0.09
	Roll	0.25	0.12	0.17	0.03	0.05	0.06
	Yaw	0.02	0.05	0.04	0.02	0.03	0.03
Penta‐Guide phantom displacement	Lat	0.09	0.11	0.18	0.02	0.04	0.03
	Lng	0.05	0.08	0.16	0.05	0.04	0.02
	Vrt	0.05	0.12	0.08	0.02	0.02	0.01
	Roll	0.06	0.10	0.16	0.02	0.03	0.03
CIRS lung phantom displacement	Lat	0.29	0.04	0.04	0.02	0.17	0.04
	Lng	0.16	0.04	0.10	0.02	0.07	0.04
	Vrt	0.08	0.04	0.08	0.02	0.04	0.01
	Roll	0.08	0.04	0.24	0.02	0.14	0.05

**FIGURE 4:**
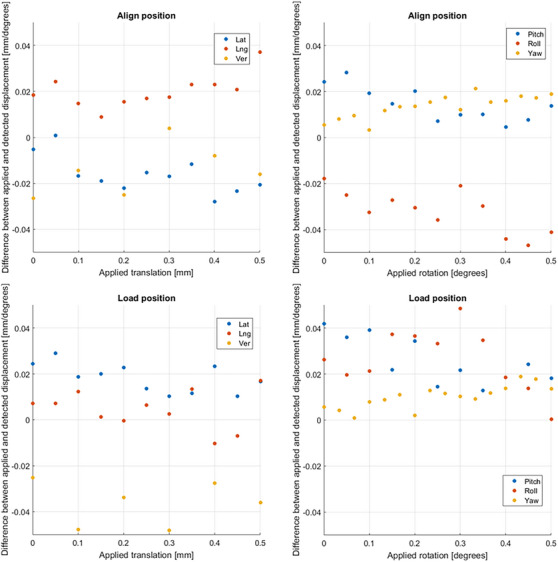
Difference between the applied and detected movement of the white head phantom, at align and load position.

Here is also shown that for a lateral translation of the CIRS lung phantom also a roll was detected. The accuracy and precision were also determined for small movements of the white head phantom (steps of 0.05 mm (lateral and longitudinal) or 0.1 mm (vertical) and 0.05 degrees (pitch and roll) or 0.033 degrees (yaw)). Figure 4 shows the mean detected value per position. The system was able to detect these changes in phantom position.

### 3.2 Reproducibility

The reproducibility was determined at the load position by translating the phantom ten times over 10 mm. The reproducibility was 0.02 mm.

### 3.3 Temporal stability

**FIGURE 5:**
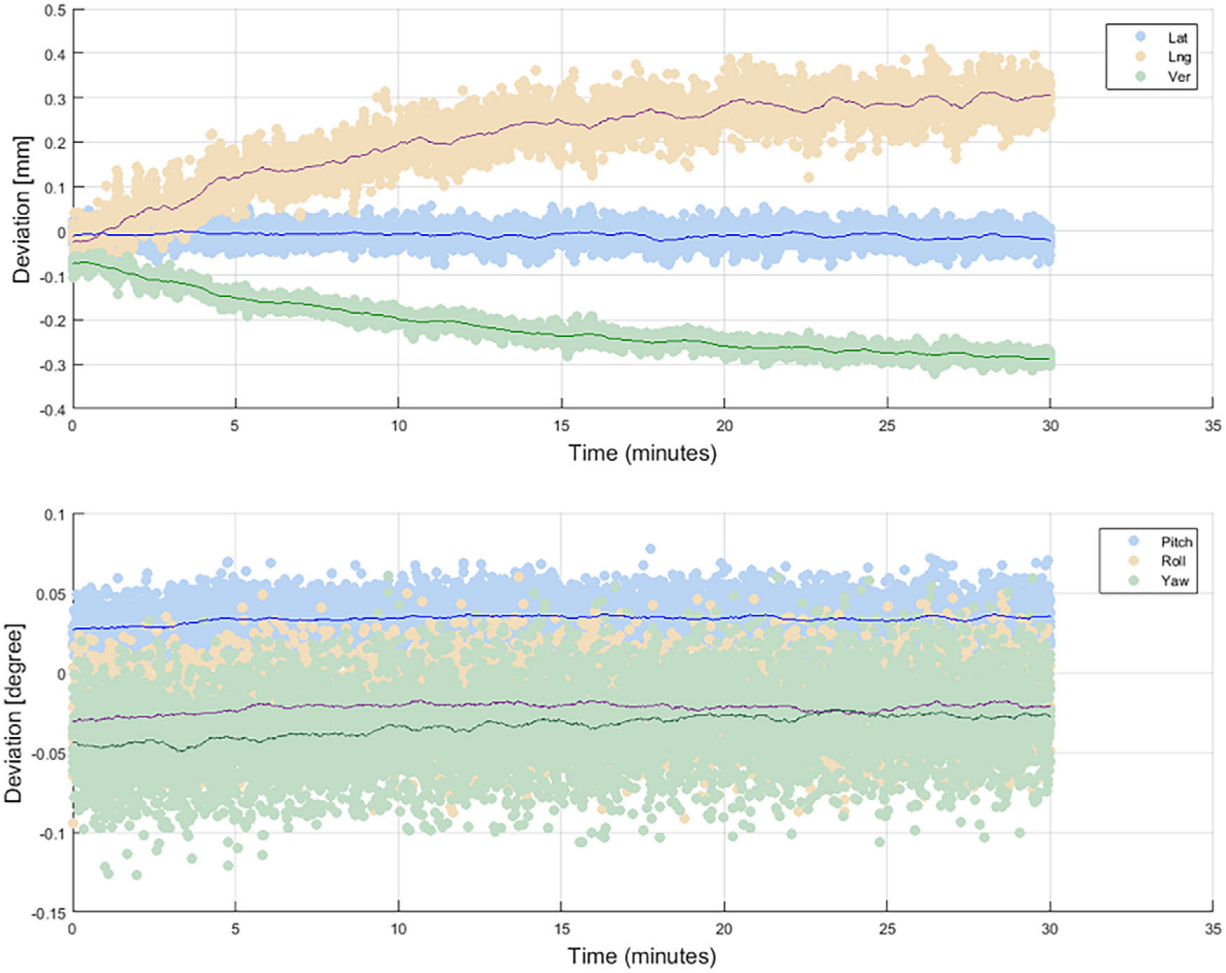
Temporal stability over a measurement of 30 min, at load position.

Figure 5 shows the translations and rotations over time. The solid lines represent the moving average over a period of 1 min. The maximum drift in load position over 30 min was 0.33 mm and 0.02 degrees in vertical direction and for yaw rotations, respectively (Table 3).

**TABLE 3:** Temporal stability of a static measurements of 30 min, at load position.

	Lat (mm)	Lng (mm)	Ver (mm)	Pitch (°)	Roll (°)	Yaw (°)
Load position	0.01	0.33	0.22	0.01	0.01	0.02

### 3.4 Effect of surface colors on localization accuracy

The accuracy of the IDENTIFY^TM^ system was slightly worse for the black and brown surface compared to the white head phantom. Table 4 shows the maximum value of the accuracy for different colors of the head phantom. For the lateral translation below 5 mm of the black phantom, also a roll rotation was detected. This effect was not observed for the brown colored surface.

**TABLE 4:** Maximum value of the accuracy of the IDENTIFY^TM^ system for different surface colors of the phantom for displacements in lateral direction, measured in load position.

	Measured deviation					
	Lat (mm)	Lng (mm)	Vrt (mm)	Pitch (°)	Roll (°)	Yaw (°)
Black surface	0.70	0.23	0.18	0.09	0.50	0.19
Brown surface	0.13	0.09	0.09	0.05	0.15	0.09
White surface	0.09	0.09	0.10	0.02	0.05	0.03

### 3.5 Effect of ambient light on the system accuracy

The ambient light level had no effect on the accuracy of the IDENTIFY^TM^ system. A slightly higher value of the precision was seen for the ambient light levels of 25 lux, however the values are still small (Table 5).

**TABLE 5:** Accuracy and precision of IDENTIFY^TM^ for different ambient light levels for displacements of the head phantom in lateral direction.

		Accuracy (mm)	Precision (mm)
25 lux	Load	0.02	0.03
	Align	0.02	0.01
70 lux	Load	0.01	0.02
	Align	0.01	0.01
90 lux	Load	0.02	0.01
	Align	0.03	0.01

### 3.6 End‐to‐end localization assessment

Table 6 shows the measured values by the IDENTIFY^TM^ system in align position, and in load position before and after the couch displacement as a result of the CBCT match. In align position, the maximum measured values by the IDENTIFY^TM^ system were 1.0, 1.1, and 0.4 mm in lateral, longitudinal, and vertical direction, respectively. In load position after the couch shift, the maximum values measured by the IDENTIFY^TM^ system were 0.6, 0.3, and 0.9 mm in lateral, longitudinal, and vertical direction, respectively. This is the localization error of the IDENTIFY^TM^ system relative to the treatment machine's radiation isocenter according to an online matched position.

**TABLE 6:** Measured values of the IDENTIFY^TM^ system on two Halcyon systems during the end‐to‐end test with the Penta‐Guide phantom.

		Halcyon system 1			Halcyon system 2	
	Align	Load, before match	Load, after match	Align	Load, before match	Load, after match
Lat (mm)	−0.6	−0.8	−0.6	1.0	−0.6	−0.5
Lng (mm)	1.1	0.5	−0.3	0.4	0.0	−0.2
Vrt (mm)	0.4	0.3	0.4	−0.4	−0.4	−0.9

### 3.7 Investigation of optimal HU threshold for skin contouring

For two patients the external body contour was created using a threshold of −400 HU, −350 HU, and −300 HU. This resulted in a local maximum difference between the contours ranging from 0.1 to 0.5 mm.

## 4 DISCUSSION

Translations and rotations of different phantoms, under various conditions, were performed to assess the performance of the IDENTIFY^TM^ system. The characterization of the SGRT system on an Ethos system and Halcyon system was performed and included measurements at the align (used for patient setup) as well as the load position (used for patient treatment). Overall, the results meet the requirements of the guidelines of the ESTRO and the AAPM and the technical performance of the system is sufficient for accurate patient setup and motion monitoring. The detected deviations of the system are clinically not relevant.

For the white head phantom the accuracy of the IDENTIFY^TM^ system was 0.07 mm and 0.07 degrees at load position, and 0.06 mm and 0.01 degrees at align position. For the Penta‐Guide phantom the accuracy in align position was comparable to the white head phantom, with 0.06 mm and 0.01 degrees. The system was less accurate for translations of the CIRS lung phantom in align position (0.20 mm, 0.05 degrees). The explanation for this might be the shape of the phantom, since the surface contains few 3D features. In the ESTRO guidelines, static accuracy was defined as the difference between applied and measured shifts or rotations and must be less than 1 mm for shifts up to 5 cm, and less than 2 mm for larger shifts. According to the guidelines of the AAPM, the accuracy should be less than 2 mm in all directions for standard dose fractionations and less than 1 mm for SBRT and SRS. Our results meet these requirements for all three phantoms.

From the analysis it was concluded that for translations of the CIRS lung phantom the IDENTIFY^TM^ system was less accurate in measuring the correct movement of the phantom. This was presented in a 6 × 6 matrix, showing the maximum value of the accuracy at a certain position. For example, when the phantom was moved in lateral direction, also a roll was measured. This can be explained by the fact that a lateral movement resembles a roll rotation that introduces a small lateral and vertical translation. If a roll is detected once, this deviation is more likely to be also detected at the next timestamp, since the IDENTIFY^TM^ registration algorithm uses the last results to initialize the computation for the next registration alignment. With the 6 × 6 matrix, the measured deviation is not only presented in the direction the phantom was moved, but also for the displacements in the other directions. The values in Table 2 are important for a complete assessment of the SGRT system. However, it is not explicitly included in guidelines on SGRT. Both ESTRO and AAPM guidelines only give recommendations on measuring the translations or rotations in comparison with the applied translations or rotations. Our results show that it is important that the measured translations or rotations in the other directions, which are supposed to be zero, are also measured. We recommend to add to the guidelines to assess the deviations in all directions and not only in the direction the phantom was moved to.

At the start of each measurement the 6 rotation and translation values were reset. At the moment of resetting, the software creates a new reference image, which is used for the subsequent measurements. At every timestamp the surface is reconstructed, by using information from the three pods. The results in Figure 3 show that although the CIRS lung phantom was not moved after resetting, the 6 values are not exactly zero. This random error was caused by the oval shape of the phantom, which has not enough topography. The plot shows that the IDENTIFY^TM^ system reported small erroneous displacements in lateral and roll direction.

Other studies have assessed the accuracy of optical surface scanning systems for a C‐arm linac. Hoisak et al. described the procedures that were performed as part of the commissioning procedure of the IDENTIFY^TM^ system.^6^ A consistency check, and a shift and rotation check were performed. The treatment couch with a resolution of 1 mm was used to displace the phantom. For the shift check, the couch was translated from 1 to 50 mm and the measured value by the IDENTIFY^TM^ system was compared to the known treatment couch shift. The accuracy of the shifts was within 0.6 mm. Our results showed a better accuracy, with a maximum value of 0.11 mm for the white head phantom. A possible explanation for this difference is that the measured accuracy in the study of Hoisak et al. was limited by the accuracy of the couch. For the rotation accuracy check the couch was rotated from 0 degrees to 75 degrees and the yaw detected by the IDENTIFY^TM^ system was compared to the known couch rotation and this was within 0.1 degrees, similar to our results of the measurements with the white head phantom. Pitch and roll were not investigated.

Zhao et al. described a method to validate the measured values reported by an SGRT system, using a six‐degree‐of‐freedom couch.^13^ They quantified the accuracy of the IDENTIFY^TM^ system on a C‐arm linac for translations and rotations of a styrofoam head phantom up to ±1.5 mm and ±0.5 degrees. Larger translations and rotations of the phantom were not assessed. Measurements were performed for different couch rotations and when one of the three pods was blocked. The median value of the magnitude of the residual translational error ranged from 0.13 to 0.61 mm for different couch rotations and increased to 0.70 mm when one pod was blocked. The lowest value of 0.13 mm was for a couch angle of 0 degrees and without pod blockage, which is comparable to our result. Considering the residual error of the rotations, the roll, pitch, and yaw ranged from −0.44 degrees to 0.34 degrees without pod blockage.

The phantom was moved ten times to the same position, to determine the reproducibility of the IDENTIFY^TM^ system. Although the guidelines of the AAPM and ESTRO do not describe such a measurement, we think it is important to evaluate if the results are comparable when the phantom is moved repeatedly. With a variation of 0.02 mm our reproducibility has a lower value compared to the results of Hoisak et al., where a reproducibility of <0.1 mm was reported.^6^

Our results show a drift in translational direction within 0.33 mm, and within 0.02 degrees in rotational direction. According to the guidelines of the AAPM, the spatial drift should be less than 2 mm in 1 h. The ESTRO guidelines require a thermal drift of 1 mm and 1 degree. Our results meet these requirements. Compared to our results, Hoisak et al. reported a similar temporal drift over 25 min continuous recording with a variation of <0.24 mm (± 0.12 mm) in translational direction.^6^ In rotational direction Hoisak et al. reported a higher value of the drift of 0.1 degrees (± 0.01 degrees).

The effect of surface color on the accuracy of the IDENTIFY^TM^ system was small and for clinical use not relevant. The accuracy of the system was slightly worse for the black and brown head phantom compared to the white head phantom. Only for lateral translations below 5 mm of the black surface, the system was not able to distinguish a lateral translation from a roll rotation. It can be concluded that the IDENTIFY^TM^ system can adequately monitor patients with different skin colors. The AAPM guidelines require to assess the effect of surface color on the localization accuracy by testing both light‐and dark‐toned phantoms.

The effect of the ambient light level for translations of the white head phantom was negligible. The ESTRO guidelines require a tolerance of 0.5 mm and 1 degree and our results meet this requirement. The ambient light level might have an effect when different surface colors are applied. This is work for further research.

The results of the end‐to‐end localization assessment show an error of the IDENTIFY^TM^ system relative to the treatment machine's radiation isocenter of maximum 0.6, 0.3, and 0.9 mm in lateral, longitudinal, and vertical direction, respectively. This result falls within the guidelines of the AAPM and the ESTRO of 1 and 2 mm, respectively. For a closed‐bore linac both the treatment machine's radiation isocenter and the localization error in align position are important. Patient positioning is performed in align position, while treatment is performed in load position. However, the description of the end‐to‐end test according to the guidelines does not mention such an align position. Our results show a localization error in align position of maximum 1.0, 1.1, and 0.4 mm in lateral, longitudinal, and vertical direction, respectively. This error includes the inaccuracy of positioning the phantom according to the laser lines.

On a Halcyon system a radiographic isocenter calibration is performed on the system, hence the results reported by the IDENTIFY^TM^ system in load position agree with the match result of the CBCT‐scan. For the current version 2.3 of the IDENTIFY^TM^ system installed on an Ethos system, no radiographic isocenter calibration process is necessary. This could result in a discrepancy between the results reported by the IDENTIFY^TM^ system and the match results of the CBCT‐scan. For newer versions of the IDENTIFY^TM^ system (3.0 and higher), a radiographic isocenter calibration is necessary and the results of the end‐to‐end test given in this paper apply.

The local maximum difference between the external skin contours based on different HU thresholds ranged from 0.1 to 0.5 mm. This is acceptable for accurate patient setup, taking the margin into account that is applied for other uncertainties. The ESTRO guidelines mention a consistent definition of surface contour to be used. This is met by applying automatic skin contouring in the treatment planning system based on a fixed HU threshold value.

On the Ethos system, patient positioning is performed at the align position and the motion monitoring during treatment is performed at the load position. Both positions are important to study, since the reconstruction of the surface is performed at a different camera angle for the different couch positions. The accuracy of the SGRT system is influenced by the angle of the surface in relation to the camera, and the distance from the surface to the camera. The yaw rotation measurements showed that the accuracy at load position was better compared to align position. This can be explained by the fact that the back camera is not used when the object is at align position. At load position, the surface is closer to the camera. However, the deviations are small enough to be able to perform accurate patient positioning and motion monitoring at align as well as load position. Note that in a clinical setting motion monitoring is only performed at load position.

An advantage of the IDENTIFY^TM^ system installed on a closed‐bore system compared to a C‐arm linac, is that camera pod blockage by the gantry, imaging panels, or kV‐tube is not possible. Pod blockage is still possible by the patient itself. Especially in clinical situations, it is important to take this effect into account, since the positioning of the patient in relation to the cameras, shape of the surface and camera blockage by the patient itself can often not be influenced during the clinical workflow. For example, the registration of a flat surface like a male thorax, containing little surface information, could be less accurate when one camera pod is blocked and the ROI is not visible for all three camera pods. A small object with many 3D features, such as the head phantom used in this study, is easier to register with the reference image, even when only the surface which is visible for one or two cameras is used. Therefore, measurements with camera blockage in this experimental setup will not be representative for a clinical situation. The effect of camera pod blockage is work for further research.

Another SGRT system intended for use with a closed‐bore linac is the AlignRT InBore. This system combines cameras mounted to the ceiling with bore‐mounted cameras for in‐bore motion monitoring. Nguyen et al. assessed the performance of a prototype of the system and reported an accuracy of less than 0.5 mm and a temporal stability of less than 0.4 mm.^14^ In a study of Flores‐Martinez et al. the accuracy of AlignRT used in combination with Halcyon was assessed by phantom measurements. They measured a rotational shift of less than 0.2 degrees.^15^ Our results showed a better accuracy of the IDENTIFY^TM^ system compared to the results of Nguyen et al. and Flores‐Martinex et al.

In this study we used a phantom to perform rigid transformations. To assess deformable transformations in detail, a deformable phantom has to be used. Palotta et al. applied such a deformable phantom to study the accuracy of the Catalyst (C‐RAD Positioning AB, Uppsala, Sweden) system.^16^ That SGRT system combines a deformable image registration (DIR) tool with a rigid registration (RR) tool. They concluded that the performance of registration was improved when using DIR over RR, for deformable deformations of the phantom. The IDENTIFY^TM^ system uses a rigid registration algorithm to match the current surface to the reference image. In this study, dynamic radiation delivery was not assessed. Measurements using a phantom that is moving in real time are worth further investigation.

It can be concluded that the IDENTIFY^TM^ system can adequately be used for patient setup and motion monitoring on a closed‐bore linac with submillimeter accuracy. The results of the performed measurements meet the requirements described in the guidelines of the AAPM and the ESTRO.

## AUTHOR CONTRIBUTIONS

Janita Dekker, Mariska de Smet, Sander van het Schip, and Willy de Kruijf designed the study. Janita Dekker and Sander van het Schip collected the data and Janita Dekker performed the analysis. Janita Dekker, Marion Essers, Mariska de Smet, Willy de Kruijf, and Martijn Kusters interpreted the data, and Janita Dekker wrote the paper. All authors discussed the results, and read and approved the final manuscript.

## CONFLICT OF INTEREST STATEMENT

The authors declare no conflicts of interest.
